# Genetic analyses and detection of point mutations in the *acetylcholinesterase-1* gene associated with organophosphate insecticide resistance in fall armyworm (*Spodoptera frugiperda*) populations from Uganda

**DOI:** 10.1186/s12864-022-09093-4

**Published:** 2023-01-16

**Authors:** Geresemu Omuut, Happyness G. Mollel, Dalton Kanyesigye, Félicien Akohoue, Stella Adumo Aropet, Henry Wagaba, Michael H. Otim

**Affiliations:** 1 National Agricultural Research Organization, National Crops Resources Research Institute, P. O Box, 7084 Kampala, Uganda; 2 Tanzania Agricultural Research Institute-Mikocheni, P. O. Box, 6226 Dar es Salaam, Tanzania; 3 Laboratory of Genetics, Biotechnology and Seed Science (GBioS), Faculty of Agronomic Sciences, University of Abomey-Calavi, Abomey-Calavi, Benin; 4 State Plant Breeding Institute, University of Hohenheim, 70599 Stuttgart, Germany; 5 National Agricultural Research Organization, National Agricultural Research Laboratories, P.O. Box 7065, Kampala, Uganda

**Keywords:** Fall armyworm, *Spodoptera frugiperda*, Corn/rice strains, Haplotypes, *Cytochrome oxidase I*, *Triosephosphate Isomerase*, *Acetylcholinesterase*, Point mutations

## Abstract

**Background:**

The fall armyworm (FAW), *Spodoptera frugiperda;* J.E. Smith (Lepidoptera: Noctuidae), is now an economically important pest that causes huge losses to maize productivity in sub-Saharan Africa. Variations in sub-population genetics and the processes of rapid adaptation underpinning the invasion remain unclear. For this, the genetic identity and diversity of FAW populations in Uganda were revealed by sequencing 87 samples (collected across the country). Based on the partial *mitochondrial cytochrome oxidase* I (*CO*I) gene polymorphisms, we further examined the mitochondrial haplotype configuration and compared the FAW in Uganda with sequences from other parts of the world. The molecular target for organophosphate and carbamate resistance, *acetylcholinesterase*, was also investigated.

**Results:**

Analysis of the partial *CO*I gene sequences showed the presence of both rice (predominant) and corn strain haplotypes, with a haplotype diversity of 0.382. Based on the *CO*I marker, pairwise difference distribution analyses, and neutrality tests, showed that the FAW populations in Uganda and the rest of Africa are evolving neutrally, but those in America and Asia are undergoing expansion. Our findings support observations that invasive FAW populations throughout the rest of Africa and Asia share a common origin. Sequencing of the *S. frugiperda ace-1* gene revealed four amino acid substitutions, two of which (A201S and F290V) were previously shown to confer organophosphate resistance in both *S. frugiperda* and several other insect species. The other two previously reported new variations in positions g-396 and g-768, are presumed to be related to the development of insecticide resistance.

**Conclusions:**

This research has increased our knowledge of the genetics of FAW in Uganda, which is critical for pest surveillance and the detection of resistance. However, due to the low gene polymorphism of *COI*, more evolutionary studies incorporating the *Spodoptera frugiperda* whole-genome sequence are required to precisely understand the FAW population dynamics, introduction paths, origin, and subsequent spread.

**Supplementary Information:**

The online version contains supplementary material available at 10.1186/s12864-022-09093-4.

## Background

The fall armyworm (FAW), *Spodoptera frugiperda* (J.E. Smith) (Lepidoptera: Noctuidae), is a devastating polyphagous and highly migratory herbivorous insect pest, that is native to the Western Hemisphere [1]. Since its first detection in São Tomé and Príncipe, Nigeria, Togo, and Benin [2], FAW has been reported in 46 (out of 54) countries in sub-Saharan Africa and 17 Asian nations [3, 4] and recently detected in Australia (https://bfvg.com.au/2020/04/06/when-was-fall-armyworm-detected-in-australia/). The pest feeds on 353 host plant species in 76 plant families [5], with a high preference for maize - a staple food crop for over 300 million African smallholder farmers [6–8]. FAW damages maize plants by voraciously feeding on leaves, stems, and reproductive parts, thus destroying their growth potential [9, 10]. The young larvae feed on young leaves, while older larvae penetrate the whorl (throat), buds, and stems [10–12]. The damage usually starts from the margin and proceeds to the midrib of the leaf creating clear perforations. In older maize plants, FAW also burrows into the ear and feeds on kernels [10, 11].

The establishment of FAW in Africa is of special concern. In 2017, 2.5 to 6 million USD maize loss was estimated in Africa [12]. The annual loss estimates of maize due to FAW in 2018 were USD 177 M and USD 159 M in Ghana and Zambia, respectively [13]. In Uganda, FAW was estimated to be capable of causing 15 and 75% yield loss of maize [14]. In only the 2017 cropping season, Uganda’s estimated loss was about USD 192 M, which directly affected 3.6 (about 10% of the population) million people [14]. Without proper control measures, the FAW was estimated to have the potential to cause annual maize yield losses between USD 2.48 billion and USD 6.19 billion in 12 African countries [15]. The invasion and rapid spread of FAW in Africa therefore, threatens the food, nutrition, and income security of millions of smallholder farmers.

*Spodoptera frugiperda* exists in two morphologically identical but genetically distinct strains: the corn strain designated as “C” strain and the rice strain designated as “R” strain, which are considered to differ in host preference, physiology, and behavior [16–19]. The corn strain is thought to be associated with maize, cotton, and sorghum, whereas the rice strain is thought to feed on rice and other pasture grasses though this is not always consistent as observed in Argentina [20]. The only accurate method of identifying these two morphologically indistinguishable strains is by the use of molecular markers [4, 20, 21]. Because the two strains of FAW have similar morphological characteristics, the mitochondrial gene *cytochrome oxidase* subunit I (*CO*I) and the nuclear gene *triosephosphate isomerase* (*Tpi)* polymorphisms are used to distinguish them [18, 22] and to study their diversity. In the Western Hemisphere, there is a significant concordance between the two markers for FAW strain identification. However, various studies have reported inconsistent results when the two markers were used to investigate the FAW strain identity from Africa, India, and Southeast Asia [23, 24]. Though some disagreements have been reported among them [18, 23–26], these are the most common and currently used markers for identification, divergence studies, and comparative analyses of FAW. According to previous studies, the *Tpi* gene can accurately predict host association [24, 27, 28]. In Africa, the observed inconsistencies between the two markers indicate the hybrid nature of invasive populations [27, 28]. Recently, some attempts have been made to identify the strains using whole-genome sequencing [4].

The fall armyworm’s remarkable environmental adaptation is reflected in its genetics and polyphagy [9] and the development of resistance to insecticides and genetically modified crops expressing *Bacillus thuringiensis* (*Bt*) toxins [29–32]. To date, field-evolved resistance to several chemical pesticides and *Bt* crops have been recorded in multiple FAW populations [33–35], resulting in severe crop production losses and extensive use of insecticides.

Losses attributed to FAW damage in maize-dependent smallholder and commercial farms across Africa have raised a need for management interventions to guard against the present and future food insecurity. The use of insecticides in emergency response to reduce FAW spread and damage has been widespread since its introduction into the continent [36]. In Uganda, the government promoted primarily organophosphates and pyrethroids to regulate FAW. Although vital for management, intensive or inadequate use of pesticides poses selection pressures on insects resulting in the development of insecticide resistance [15]. The reported ineffectiveness of the synthetic Organophosphate and pyrethroid (lambda-cyhalothrin) insecticides by many African farmers, necessitated the use of higher doses of the insecticides to get adequate penetration and kill larvae feeding deep within the whorl of the plants. Although *S. frugiperda* has been reported in Uganda, there is limited knowledge on its genetic diversity, distribution, and resistance status. The existing information in Uganda indicates the occurrence of two strains, identified using *COI* markers [22]. Moreover, only a small number of samples from just four districts have been studied using both *COI* and *Tpi* markers [26], and few locations for the occurrence of resistance mutations [37]. Limited knowledge on the genetic structure and resistance profiles and lack of comparative genetic information, have limited advances to explore the biogeographic patterning, and the relatedness of FAW haplotypes in Uganda with those of other geographic locations of the world. We therefore, conducted analyses to document genetic and insecticide resistance status of FAW using the *COI, Tpi*, and *ace-1* partial genes DNA markers. Of key interest, analyses of genetic identity, structure and diversity of FAW populations in Uganda were conducted. We also analyzed whether the invading FAW carry point mutations associated with resistance to organophosphate insecticides within the *acetylcholinesterase* 1 gene.

## Results

### PCR amplification of partial gene segments, and molecular identification of *Spodoptera frugiperda*

All primer sets used in this study amplified successfully and gave PCR products of *COI*A, *COI*B, *Tpi,* and *ace*-1 genes of the expected band sizes (supplementary Fig. 1A-1D). Of the 95 *S. frugiperda* samples analyzed, 3 samples did not amplify with PCR. The 92 samples which amplified were sequenced. Out of the 92 sequences (for both *COI* and *Tpi* markers), 5 sequences (which were poor in quality) were not considered in downstream analyses. Only 87 sequences were therefore used for genetic diversity and resistance studies.

**Fig. 1:**
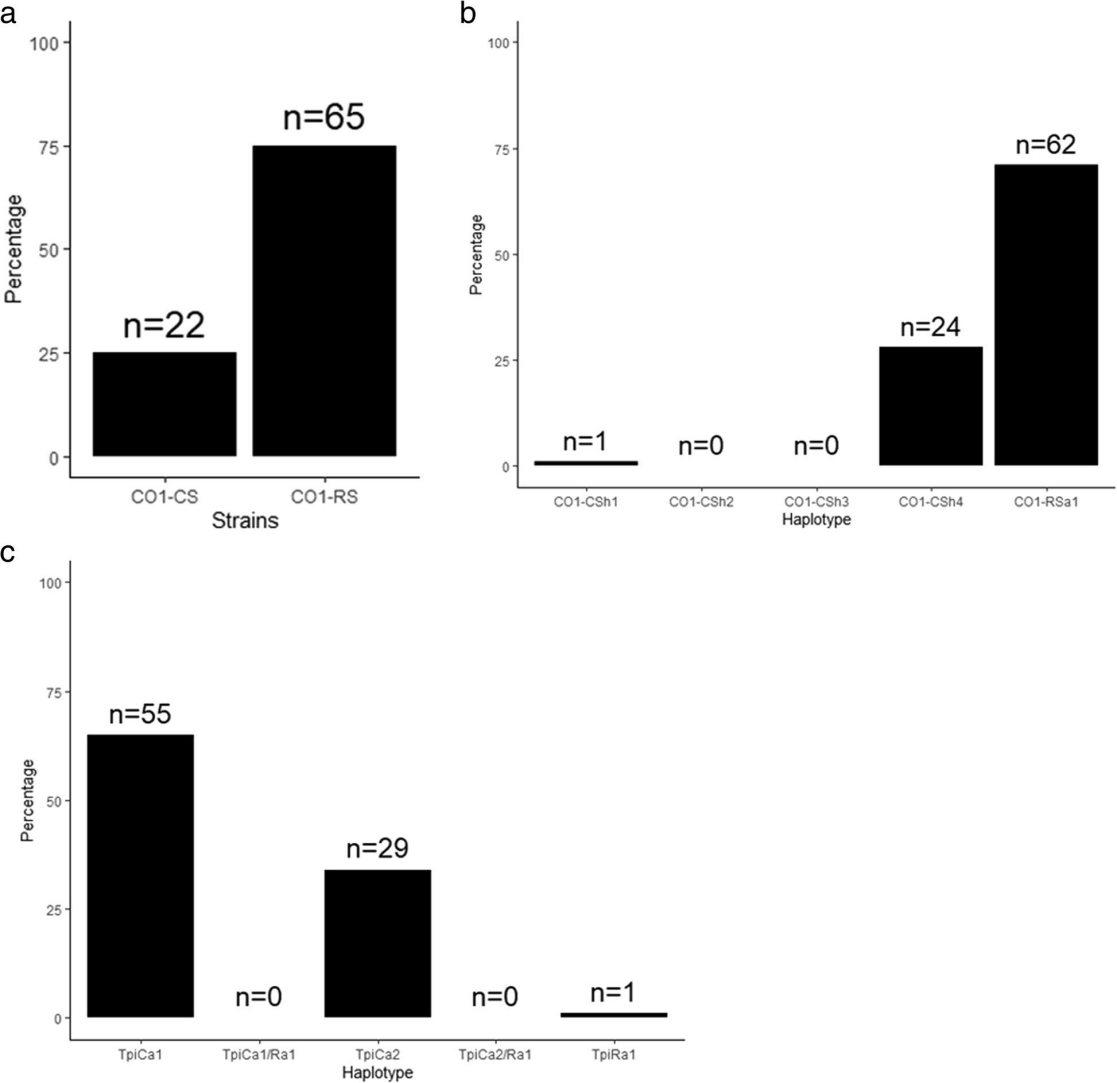
Strain distribution pattern for *Spodoptera frugiperda* populations in Uganda based on mitochondrial *CO*I and *Tpi* partial gene markers. **a ***CO*IA distribution; **b ***CO*IB distribution; **c ***Tpi* distribution

The investigation of the *mCO*I602 strain defining polymorphic locus found in the barcode region (*COI*A), revealed the “R” strain to be found in 75% (*n* = 65; Fig. 1a) of the 87 *COI*A sequences from Uganda, while the “C” strain was found in the remaining 25% (*n* = 22; Fig. 1a). Two GenBank accessions from this work (OP020715 and OP020716) are used to represent the *COI*A corn strain and *COI*A rice strains, respectively.

Another strain marker segment (*COI*B) was studied, which contains polymorphic loci *mCO*I1164D and *mCO*I1287R, resulting in five haplotypes, four of which belong to the Corn strain (CSh1–4) and one to the Rice strain category. *COI*B haplotyping was done for 87 samples of FAW from Uganda, out of which three haplotypes (which represent the entire population) were detected. Out of the three haplotypes, 71% (*n* = 62; Fig. 1b) of the samples belonged to the ‘R’ strain haplotype (*COI*-RSa1). The *COI*-CSh1 haplotype (Fig. 1b) was found in one sample (GenBank accession (OP020882), marking the first detection of the Western Hemisphere minority “C” strain FAW haplotype in Africa. The *COI*-CSh4 haplotype was found in the remaining 27% (*n* = 24; Fig. 1b). No sample belonged to the other two ‘CS’ haplotypes. The *COI*B results were consistent with those of the *COI*A barcode region marker in strain identification. The GenBank accession numbers (OP020881 and OP020883), denote the *COI*-RSa1 and *COI*-CSh4 haplotypes, respectively.

Studies on the polymorphisms at strain-defining loci of the fourth exon in the *Tpi* gene for Ugandan populations (*n* = 85), revealed three haplotypes, the *Tpi*Ca1a homozygous (*n* = 55; Fig. 1c), *Tpi*Ca2a homozygous (*n* = 29; Fig. 1c), and *Tpi*Ra1a homozygous (*n* = 1; Fig. 1c). The heterozygous corn-strain haplotypes were not found in any sample. *Tpi*Ca1a is the most prevalent haplotype among Ugandan FAW populations. *Tpi* ‘R’ haplotype was detected from Pallisa district in Eastern Uganda and shared sequence with the unique *Tpi*Ra1a haplotype which was previously detected in Africa [21]. The three *Tpi* haplotypes which were detected in this study are represented by GenBank accessions; OP185931 (*Tpi*Ca1a), OP185932 (*Tpi*Ca2a) and OP185933 (*Tpi*Ra1a). The agreement of both *COI* and *Tpi* markers would be a stricter criterion for strain identification and host association. In Uganda, however, 99% of the samples had discordant *Tpi* and *CO*I markers results for both identity and host association prediction. *Tpi* ‘C’ haplotype was detected in 99% of the samples collected from corn plants. Notably, one *Tpi* ‘R’ haplotype was also found in a cornfield, making it uncertain if *Tpi*Ra1a FAW prefer rice strain host plants or not.

### Haplotype diversity of *Spodoptera frugiperda* from Uganda

Our analysis revealed 16 polymorphic sites (16 mutations) with a nucleotide diversity (Pi) of 0.00794 after analysis of a 770-bp *CO*IA barcode region for the 87 Ugandan samples (Table 1). With a haplotype (gene) diversity (Hd) of 0.382 (Table 1), we identified two distinct haplotypes (the corn and rice strains) from Ugandan *CO*IA sequences. The majority of the *CO*I sequences (75%; n = 65) studied belonged to a single rice haplotype (Uganda _haplotype 1, represented by GenBank accession number: OP020716) which was found across the country. The rest (n = 22) belonged to the ‘C’ strain haplotype (Uganda_ haplotype 2, identified with the GenBank accession number: OP020715), which was the most common. There was no region-specific haplotype in Uganda. They all showed an irregular distribution pattern throughout the country.

**Table 1:** Genetic diversity of *Spodoptera frugiperda* populations from four geographical areas (Uganda, Rest of Africa, America, and Asia) examined using the partial *mtCO*IA gene

	Uganda	Rest of Africa	America	Asia	Total
No. of sequences	87	146	99	178	510
No. of sites	770	545	545	545	545
No. of polymorphic sites	16	11	28	33	52
No. of mutations	16	11	29	33	54
No. of haplotypes	2	5	19	19	39
Haplotype diversity	0.382	0.302	0.667	0.480	0.465
Nucleotide diversity	0.00794	0.00460	0.00898	0.00464	0.00607
Fu’s Fs statistic	20.692	5.095	−1.593	−4.616	− 15.376
Fu and Li’s D* test statistic	1.61827	−0.00493	−3.84904	−5.08159**	−9.08877**
Fu and Li’s F* test statistic	2.36745	0.27336	−2.99994	−4.37879**	−6.72245**
Tajima’s D	2.63822	0.66175	−0.38805	−1.61589	− 1.62264*

(**P < 0.02) (*P < 0.05), No. =number

**Fig. 2:**
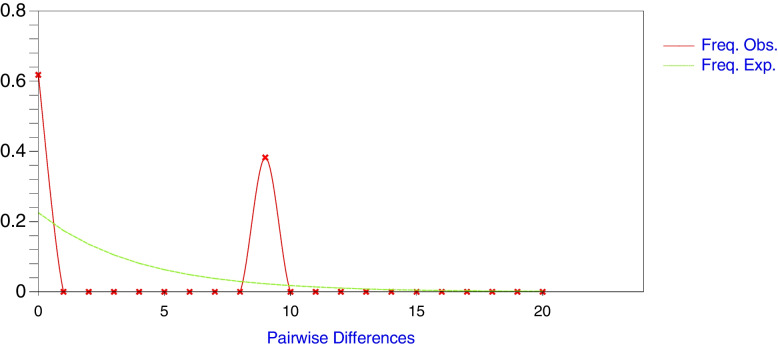
The *COI*A mismatch distribution curve showing the observed (solid red line) and expected (dotted green line) pairwise nucleotide site divergences computed with DnaSP

The results of Fu’s Fu test and Tajima’s D test statistic were both significantly positive (Table 1). Within the ‘R’ strain (n = 65) and ‘C’ strain (n = 22) populations, there was no nucleotide diversity detected. In Uganda’s FAW population, there was no evidence of population expansion, and the mismatch distribution for FAW strains showed a bimodal curve, indicating population neutral evolution (Fig. 2).

### Comparative genetic analyses of Ugandan *Spodoptera frugiperda* population and other geographic regions

**Fig. 3:**
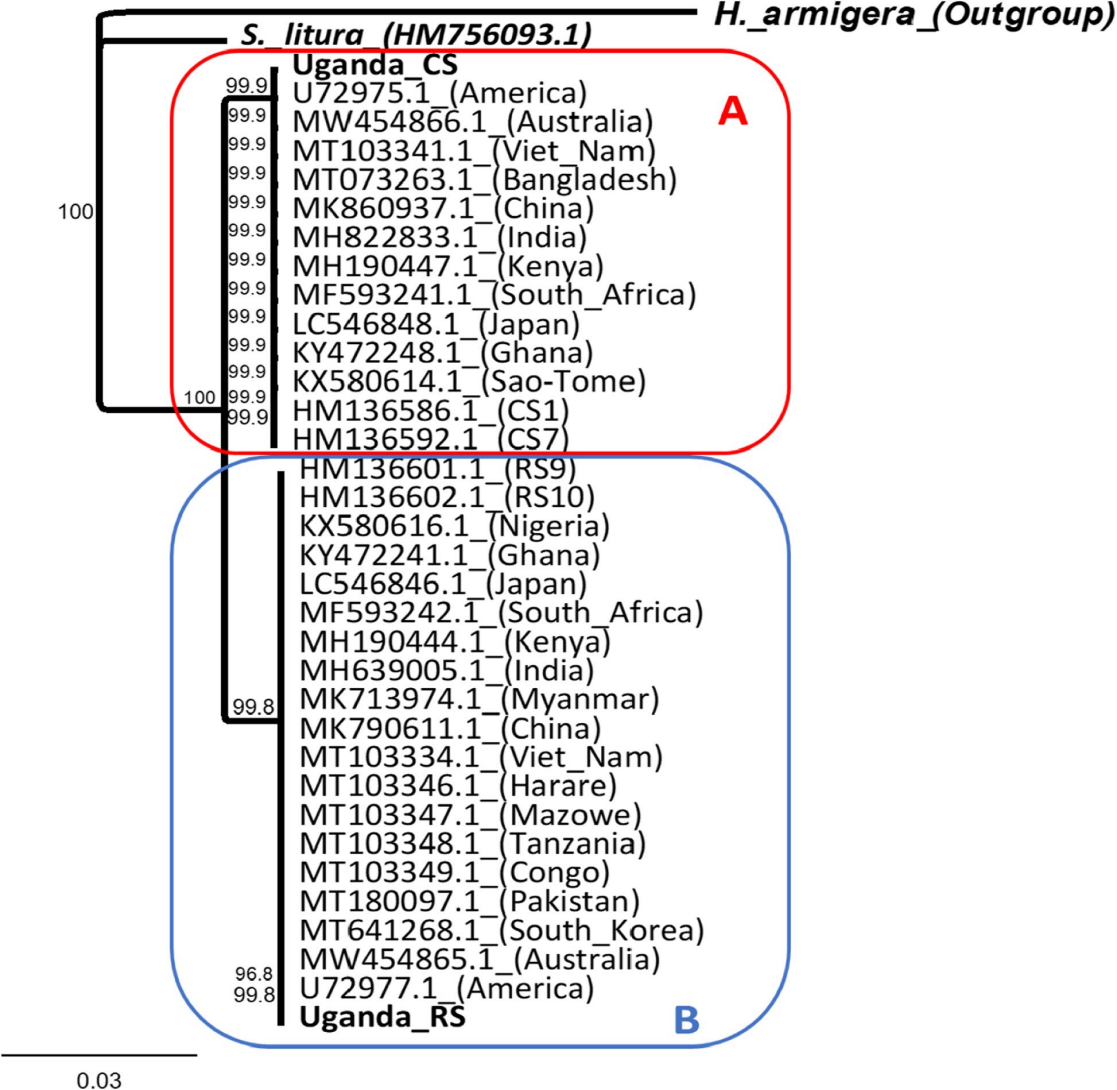
Consensus phylogenetic tree derived from a neighbor-joining analysis comparing the two Ugandan *CO*I haplotypes with those from *Spodoptera frugiperda* host strains of other countries and one related *Spodoptera* species [38]. *Spodoptera frugiperda* haplotypes CS1 and RS9 are prevalent fall armyworm haplotypes in the Western Hemisphere. As an outgroup, *Helicoverpa armigera’s CO*I barcode section was used. The jukes-Cantor genetic distance model was used in building the tree. At branch points, 1000X bootstrap values are represented as numbers. The substitutions per site are depicted by the scale bar. Each species is accompanied by its GenBank accession number

**Fig. 4:**
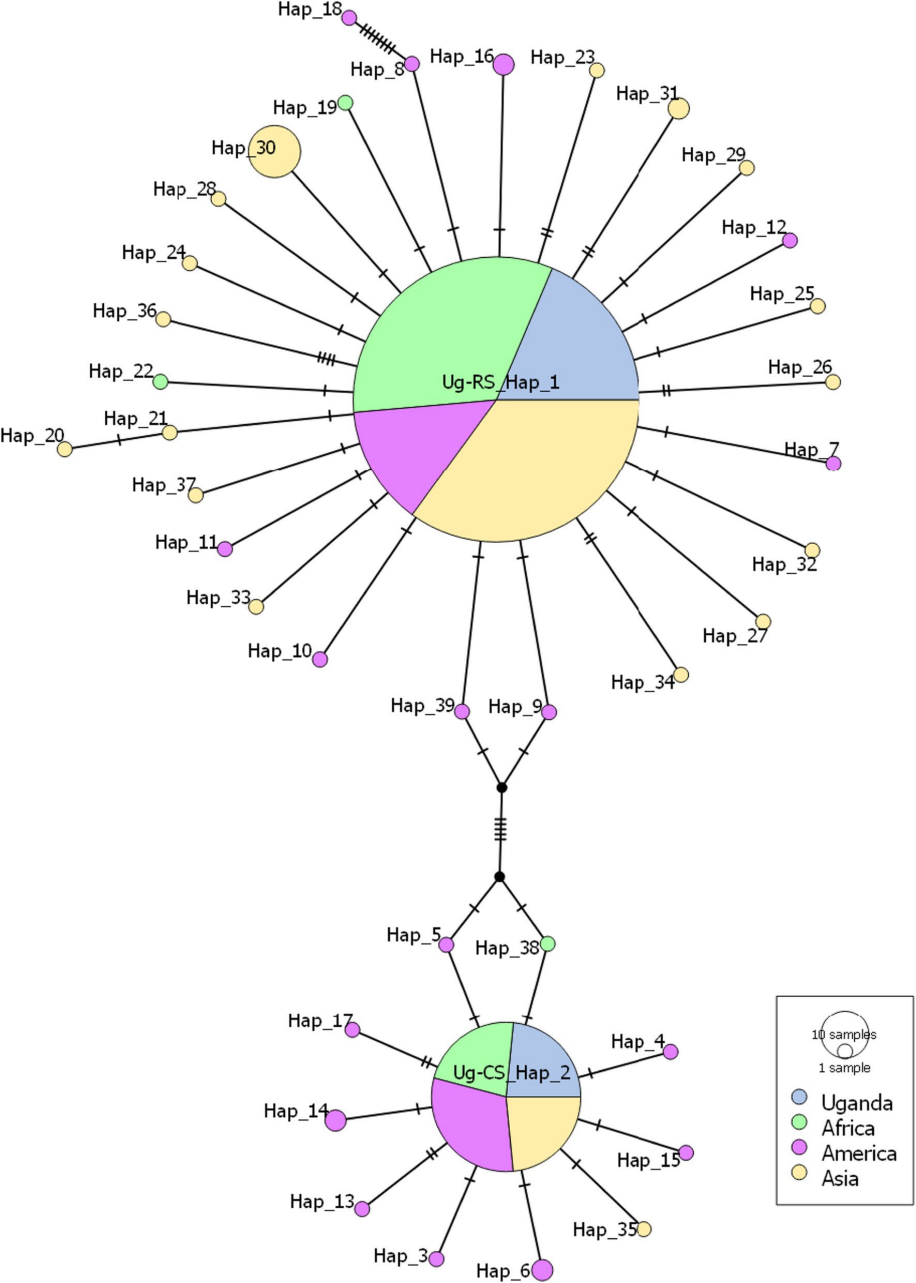
Median-joining haplotype network of *Spodoptera frugiperda mtCO*IA gene partial sequences from four geographical groups viz., Uganda, Africa, America, and Asia. Each pie represents a distinct haplotype, with the radius equal to the number of sequences associated with that haplotype. The haplotype representation among the four geographical groups is represented by the pie’s divisions. The network’s edge tallies represent the number of mutations that separate the haplotypes

Comparative investigation of a 545 bp *CO*I partial gene region that was common to all of the sequences, revealed 5, 19, and 19 haplotypes from the rest of Africa, America, and Asia, respectively (Fig. 4). The dominant ‘R’ and ‘C’ *CO*I haplotypes from America (GenBank Accession: U72977.1, U72975.1) were identical to Uganda RS and CS, respectively (Fig. 3), and represent the two major haplotypes in all invaded locations. Apart from the major haplotypes, the rest of Africa, America, and Asia were represented by 3, 17, and 17 unique region haplotypes, respectively. Compared to other regions, neutrality test statistics for Ugandan and the rest of African populations indicated that populations in these areas are still evolving in a neutral manner (Table 1). Phylogenetic comparisons revealed that one distinct RS from Uganda was identical to haplotypes from America, Asian nations, and other African countries (Fig. 3 clade B), and the same was the Uganda CS haplotype (Fig. 3 clade A).

### Haplotype network analysis

The median-joining haplotype network revealed two *CO*IA FAW rice and corn strains haplotypes (Ug-RS_Hap_1 and Ug-CS_Hap_2, respectively) found in Uganda (Fig. 4). Each of the four geographical regions shared both haplotypes. The prevalence of novel haplotypes in three regions (Africa, America, and Asia) however, differs significantly. While there are 19 distinct rice/corn haplotypes in Asia, rice strain haplotype is the most prevalent in the two invaded regions, with 3 and 17 novel haplotypes in Africa and America, respectively (Fig. 4). All of the invaded locations, according to the network, have two maternal lineages (Fig. 4). Aside from that, there is no evidence to suggest several introduction events in the invaded territories based on the *CO*IA marker information. The majority of novel corn and rice haplotypes were discovered in America and Asia, respectively (Fig. 4).

### The genetic structure of *Spodoptera frugiperda* populations

The PhiPT values were insignificant in all the four locations, indicating no genetic differentiation (Table 2). There was more genetic variation (89%) within than among (11%) the four geographical groups (Table 2). Furthermore, no differences were found between native American and invasive populations with Ugandan groups. The same observations were made for the rest of Africa and American native invasive populations.

**Table 2:** Analysis of Molecular Variance (AMOVA) results for the four *Spodoptera frugiperda* geographical groups

Groups	Source	Df	SS	MS	Est. Var.	Total variance (%)	PhiPT	*P-* value
All	Among populations	3	17.256	5.752	0.357	11	0.109	0.191
	Within populations	35	101.873	2.911	2.911	89		
	Total	38	119.128	8.663	3.268	100		
Uganda and Asia	Among populations	1	3.302	3.302	0.325	13	0.132	0.196
	Within populations	17	36.382	2.140	2.140	87		
	Total	18	39.684	5.442	2.465	100		
Uganda and America	Among populations	1	0.203	0.203	0.000	0	−0.359	1.000
	Within populations	17	63.324	3.725	3.725	100		
	Total	18	63.526	3.928	3.725	100		
Rest of Africa and Asia	Among populations	1	4.345	4.345	0.278	11	0.113	0.112
	Within populations	20	43.882	2.194	2.194	89		
	Total	21	48.227	6.539	2.472	100		
Rest of Africa and America	Among populations	1	1.131	1.131	0.000	0	−0.097	0.940
	Within populations	20	70.824	3.541	3.541	100		
	Total	21	71.955	4.672	3.541	100		
Asia and America	Among populations	1	12.992	12.992	0.563	16	0.163	0.011
	Within populations	34	98.508	2.897	2.897	84		
	Total	35	111.500	15.890	3.460	100		

### Detection of target site point mutations associated with organophosphate resistance in *Spodoptera frugiperda* populations from Uganda

**Fig. 5:**
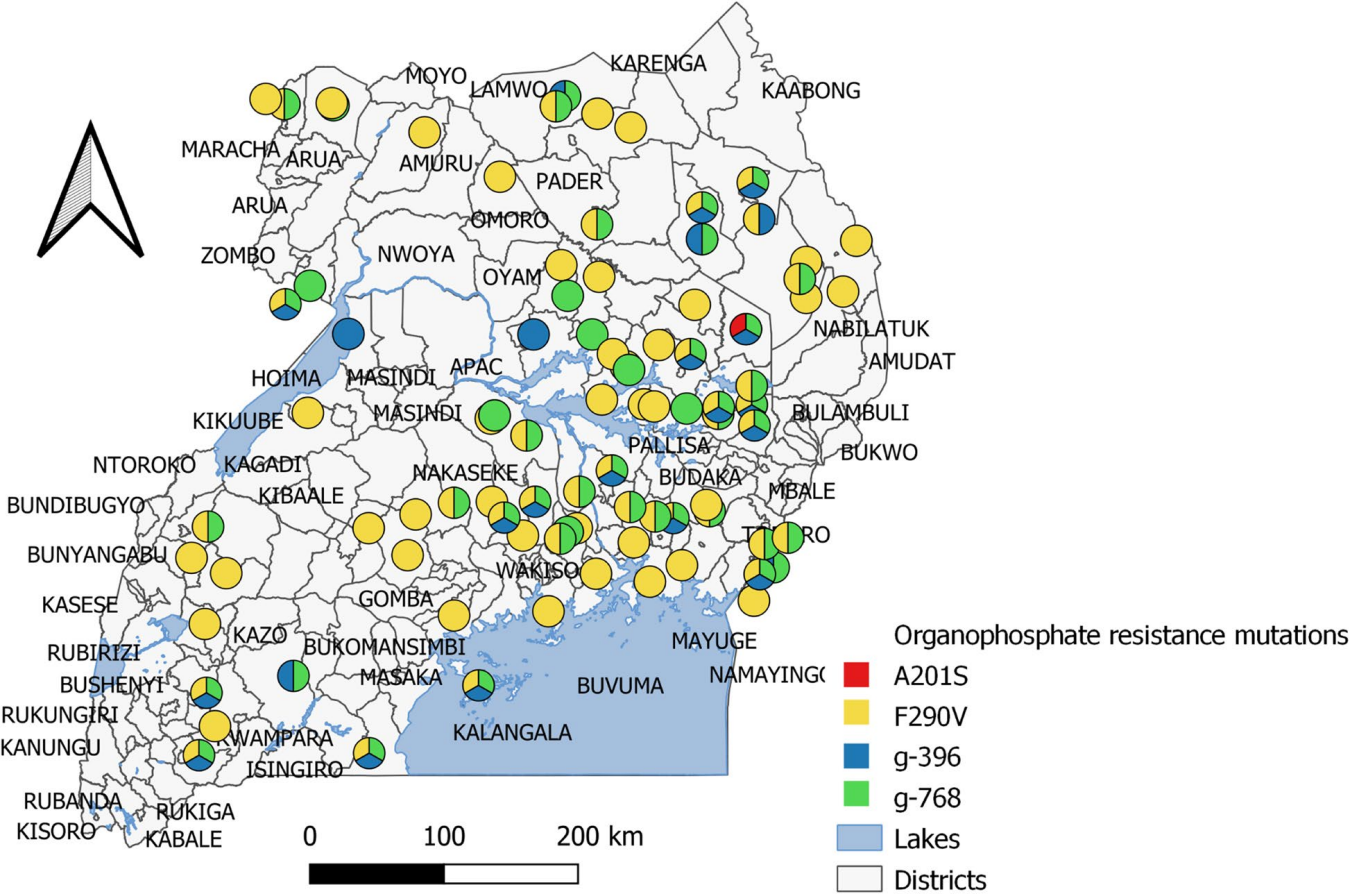
Map of Uganda showing the distribution of the *ace*-1 point mutations detected in this study. Point mutations (A201S, F290V, g-396 and g-768 are colored red, yellow, blue, and green respectively)

We identified two candidate point mutations in the *ace*-1 gene in our samples (Fig. 5). The F290V mutation was detected in samples from all regions of Uganda (mean allele frequency = 0.73). The A201S mutation was present in only one region (mean allele frequency = 0.008). A highly conserved point mutation site (G227A) [38, 39] in the position in *acetylcholinesterase* was not identified in our samples. Two of the three *ace*-1 point mutations (A201S and F290V) were detected together in a single insect and the F290V allele was the most common point mutation detected across the country (Fig. 5).

We also analyzed the other three reportedly new variations in positions (g-396 G/A, g-498 A/G, and g-768 C/G) which could be related to the development of resistance [40]. The results showed mean allele frequencies of 0.23, and 0.45 for the g-396, and g-768 point mutations, respectively, whereas the g-498 was not found in samples analyzed in the study. The list of the representative GenBank sequences used for ace-1 point mutation detection in this study include; OP185934-OP185937.

## Discussion

The fall armyworm has become a new economically important pest of maize in sub-Saharan Africa [41]. An earlier study that analyzed the diversity of Ugandan FAW populations was performed with samples from a few locations of the country [22] and only identified the corn and rice strains. It was necessary therefore to understand the diversity of the FAW populations across the country after the invasion in 2017. This paper reports on the genetic structure, diversity and distribution, the existence of corn sub-haplotypes, comparative analysis, and the detection of organophosphate point mutations in the FAW populations invading Uganda. Two strains were identified in Ugandan populations based on mitochondrial *CO*IA gene polymorphisms, with 75% of the samples belonging to the *CO*IA ‘R’ strain and the rest to the *COI*A “C” strain. Similar findings were also reported by [22]. Furthermore, a similar *COI*-RS predominance trend was previously documented in India, East Africa, and South Africa [24].

Another mitochondrial strain marker segment (*COI*B) was studied. This segment contains polymorphic loci *mCOI1164D* and *mCOI1287R*, resulting in five haplotypes, four of which belong to the Corn strain (CSh1–4) and one to the Rice Strain category. The relative distribution of *CO*IB haplotypes (CSh4 and CSh2) has been used to analyze FAW groups’ ancestry in America. CSh4 haplotype predominates in FAW populations from Florida and the East coast of America, whereas CSh2 haplotype predominates in FAW populations from Texas and most other regions of America [18, 40]. Upon analysis of these gene polymorphisms, a majority (*n* = 24) of the corn samples belonged to the *COI* sub-haplotype 4 (*COI-*CSh4) - which was previously reported in Togo [19] and more recently in Myanmar, India, and southeastern Asia [24]. Based on this marker, the similarity of our samples to those found in Florida and other locations suggests that the corn strain FAW populations invading Uganda and other African countries have a recent and common origin with those found in Florida and the Caribbean. However, due to the low polymorphism of *COI* gene segment, more evolutionary studies incorporating the *Spodoptera frugiperda* whole- genome sequence are required to understand the FAW population dynamics, introduction paths, origin, and subsequent global spread. We also detected the *COI*-CSh1 sub-haplotype in one of the samples analyzed, marking the first detection of the Western Hemisphere minority “C” strain FAW haplotype in Africa. As a result, it’s unknown if this Western Hemisphere minority haplotype will remain infrequent in Africa, as it is in the Americas. However, favorable agro-ecological conditions of sub-Saharan Africa [41] are likely to favor the establishment of *S. frugiperda* as an endemic pest for several generations and this could potentially favor hybridization between *S. frugiperda* strains as evolutionary pressures continue to shape and favor their reproduction, adaptation, subsequent survival, and further spread.

Although the rice and corn strains are known to associate with their hosts, [25, 42, 43], both strains were recovered in maize. Similarly, *COI*-RS haplotypes were detected in the majority of maize strain host plant collections in Myanmar recently, confirming previous findings [24]. As previously noted by [27], this could be due to the plasticity in plant choice or the markers’ incapacity to precisely and reliably discriminate between these strains. Nonetheless, the results of this study corroborate prior reports that the association between rice and maize strains and their hosts is not absolute [20].

The population genetic test statistics, based on the *CO*IA marker showed that populations in Uganda and the rest of Africa are still evolving neutrally, while populations in America and Asia are expanding. Therefore, our results suggest a possibility of one or more Asian nation (s) to be the new hotspot for the expansion of FAW *CO*IA rice haplotypes in addition to America. This observation is in line with recent findings that the FAW population in India is still expanding [44]. We found no novel region-specific haplotype(s) in Ugandan FAW populations after studying mitochondrial *COI*A gene polymorphisms, and this could support the current neutral evolution, in addition to the observed lower diversity.

We also sequenced a portion of the sex-linked *Tpi* nuclear gene that is known to distinguish between strains of FAW and detect potential cases of hybridization [18, 22, 45], and observed a 99% identification disagreement between *COI* and *Tpi* markers-a pattern which was also seen in India, South Africa, and East Africa [24, 26, 36]. Much as this result is not completely new [19, 46], the discrepancy observed in this study is remarkable because all samples (except one) identified by the *COI* marker as rice or corn strains were classed as *Tpi*-corn strains (*Tpi*- CS). The results suggest the better *Tpi* marker accuracy in determining host association and further demonstrate the possibility of a massive FAW hybridization pattern occurring in the region. However, it is worth noting that one *Tpi* ‘R’ haplotype was also found in a cornfield in Pallisa district in Eastern Uganda. As a result, it’s critical to figure out if *Tpi*Ra1a samples have a distinct feeding behavior that could have major ramifications for pest management in Africa’s crops.

Our study of six mutations previously identified to be involved in resistance to organophosphates insecticides, revealed the F290V allele as the most common (73%) across the country, while the A201S (1%) occurred at a very low frequency detected only in combination with the F290V allele, and was never found as a single mutation in a single insect. The low frequency of the A201S allele in all of the invading populations studied could suggest that the majority of these resistant FAW had only the F290V resistance mutation. The G227A allele was not identified in our samples. This mutation is highly conserved among the *AChE* family [38, 39] and thus, this characteristic could explain the absence of variation in the Ugandan FAW populations analyzed.

Whereas the allelic frequency distribution pattern in this study is different from that of Brazil (G227A = 67.5%, F290V =32.5% and A201S =17.5%), our results are similar to the pattern observed in previous samples from Uganda, China and Malawi [37]. A similar allelic frequency predominance pattern of two alleles (termed by [40] as g-301 and g-565 for A201S and F290V, respectively) was observed in the state of Zacatecas, Mexico [40]. Thus, indicating that in all the samples analyzed in these regions in the previous studies, only two alleles (A201S and F290V) were present with the F290V allele observed more frequently.

The A201S mutation which changes the amino acid alanine for serine have been reported to form hydrogen bonds that interact with the substrate, stabilizing the enzyme-substrate complex, and causing changes in the active site that modify sensitivity to the enzyme inhibiting the bond with organophosphate insecticides, thereby creating resistance [39, 46]. Its insensitivity to a wide range of carbamates and organophosphates was first reported in the *Aphis gossypii* [47]. In *Chilo suppressalis,* sequence analysis found an amino acid mutation which revealed a strong correlation between frequencies of the A201S mutation and phenotypic levels of resistance to triazophos organophosphate insecticide [48]. The previous report on *Musca domestica* [49] indicates that the insecticide resistance is significantly enhanced when the G227A and F290Y mutations occur in combination, than when each of them occurs singly [49]. In this study, the occurrence of the A201S mutation in combination with the F290V mutation in a single insect is worth noting. Whereas this was observed in 1% of the samples analyzed, a possibility of future hybridization of insects, and continual abuse of insecticides could pose selection pressures on insects thereby significantly enhancing resistance in Uganda.

The mutation F290V (reported by [40] as g-565) is documented to be associated with the increased affinity of *AChE* enzyme and Ach to increase resistance to 37 times [40]. The mutation occurs when phenylalanine is changed for valine, reducing the availability of space on the binding site of acyl with the organophosphate [40]. The AF290V mutation reportedly conferred a low resistance (6.7-fold) to organophosphate azinphos-methyl in the codling moth, *Cydia pomonella* but registered a high level of resistance (130-fold) to the carbamate carbaryl [50]. Detection of the AF290V mutation at a higher frequency (73%) across all sampled regions in the present study, could imply that the ineffectiveness of the current synthetic organophosphates insecticides used in Uganda, is chiefly due to the AF290V point mutation. Although various evidence suggests that three mutations, A201S, G227A, or F290V could singly confer resistance or enhance resistance in combination [16, 47, 51], it is not well understood whether or not the *ace*-1 mutations were carried into Uganda and Africa continent at the time of the invasion. A possibility of introduction of these mutations into Africa from the New World where FAW is native was suggested by Guan et al. [37]. However, a recent study by Yainna [52] tested whether or not the invasion was accompanied by the spread of resistance mutations from native populations, and their results supported strong selective pressure which is attributed to the higher proportion of multiple resistance mutations within *acetylcholinesterase* gene [52]. Thus, possibly, these point mutations are a result of rapid local selections in addition to being carried from the New World. This observation may be supported by the fact that since the introduction of FAW [36], synthetic pesticides have been used continuously in Uganda and Africa to minimize FAW spread and damage. According to our results, it could therefore suggest that the majority of the invasive *S. frugiperda* populations underwent rapid selection in favor of the F290V mutant allele in Uganda, and other African nations where a similar allelic frequency pattern occurs, with a few individuals selected in favor of the A201S mutant allele.

We also analyzed three other reportedly new variations in positions (g-396 G/A, g-498 A/G, and g-768 C/G) in *S. frugiperda* which could be related to the development of resistance [40]. This study detected two of the three mutations g-396, and g-768 which represented 23 and 45%, respectively. Although reported as potential SNP’s for resistance in *S. frugiperda* [40], no additional studies have been carried out so far to determine whether or not these mutations results in resistance. Such studies would be necessary for monitoring changes within the *S. frugiperda ace*-1 gene and the subsequent spread of the resistant invasive species of *S. frugiperda*.

## Conclusions

This study provides the most fundamental information on the genetic identity and diversity of *S. frugiperda* populations in Uganda. We detected two FAW groups, later identified as corn and rice strains, with the latter being more prevalent based on the *COI* marker. *Spodoptera frugiperda* in Uganda had point mutations in the *ace*-1 partial gene that have previously been documented to confer organophosphate resistance in both *S. frugiperda* and several other insect species. According to population genetic test results, populations in Uganda and the rest of Africa are still evolving neutrally, while populations in America and Asia are expanding. Both *S. frugiperda* strains are widely distributed in Uganda, and our study demonstrated that the corn strain FAW populations invading Uganda and other African countries are similar to those found in Florida and the Caribbean. However, due to the low gene polymorphism of *COI*, additional evolutionary studies incorporating the *S. frugiperda* whole genome sequence are required to comprehend the FAW population dynamics, introduction paths, origin, and subsequent global dissemination. When the mitochondrial and *Tpi* marker results were compared, the results indicated that the *Tpi* marker was more accurate in determining host association. Point mutation analysis of the *ace-1* partial gene segment revealed four mutations (A201S, F290V, g-396, and g-768) that were previously reported as being associated with resistance to organophosphate pesticides. This work has improved our understanding of pest genetics in Uganda, which is important for pest surveillance and detection of resistance. The findings of this study can be used to improve integrated management strategies aimed at the long-term development and deployment of environmentally friendly genetically modified *Bt.* corn, as well as the selection of effective insecticides for control, in combination with traditional host plant resistance breeding and other biological control methods. Crop protection, enhanced maize production, food security, and improved income for stakeholders, particularly smallholder farmers in Africa and elsewhere, will all result from effective management.

## Materials and methods

### Collection of *Spodoptera frugiperda* larvae and adult moth samples

The larval and adult samples were collected during the country-wide survey which was conducted between August and October 2017 in ninety-five (95) maize-growing districts of Uganda. All agro-ecological zones in the country were represented in the sample collection (http://www.fao.org/agriculture/seed/cropcalendar/aezones.do?isocode=UGA). About 8–9 larval and adult samples were collected from each of the 11 agro-ecological zones, resulting in a total sample size of 95. Upon field inspection, samples (both larvae and adults) were handpicked and stored individually in 5 mL screw-cap tubes containing absolute ethanol. The samples were packed in Ziploc® bags and then transported to the National Crops Resources Research Institute (NaCRRI), Namulonge molecular laboratory, and kept in -20 °C before DNA extraction.

### DNA extraction

Total genomic deoxyribonucleic acid (DNA) was extracted from 95 samples using a modified Chelex-100 method [53] previously reported by [22]. Before DNA extraction, each sample was rinsed twice with sterile molecular grade water (Thermo Fisher Scientific, UK) to remove the ethanol used for preservation. Using a clean sterile surgical blade, a leg or pseudo-leg (for adult moth or larvae respectively) of each sample was excised and placed in a sterile 1.5 mL micro-centrifuge tube. Fifty microliters of 10% Chelex 100 solution were added followed by 10 μL (20 mg/mL) of Proteinase K solution. Each sample was incubated at 56 °C overnight, followed by a brief vortex and heat inactivation at 100 °C for 15 minutes. The samples were then centrifuged at 15,900 relative centrifugal forces (rcf) for 3 minutes. Forty microliters of supernatant (genomic DNA) of each sample were collected into a new sterile 1.5 mL microfuge tube and stored at -20 °C until PCR amplification. The unused portions of the samples were stored in absolute ethanol and stored at -20 °C.

### PCR amplification and sequencing of *COI*, *Tpi,* and *Ace-1* partial genes of *S. frugiperda*

Partial gene sequences of *COI* and *Tpi* were amplified using three different primer sets, previously designed and used by [19]. To amplify the FAW *COI*A barcode region, primer pair (*COI*_101F/ *COI*_911R), *COI*_101F, 5′-TTCGAGCTGAATTAGGGACTC-3′ and *COI*_911R, 5′-GATGTAAAATATGCTCGTGT-3′ that give amplicons length of 811 bp, were used (Supplementary Fig. 2A). To identify *S. frugiperda* haplotype profiles and confirm FAW species host strain identity in Uganda, a 603 bp fragment of the *COIB* gene region was amplified with *COI*_ 891F, 5′-TACACGAGCATATTTTACATC-3′, and *COI*_1472R, 5′-GCTGGTGGTAAATTTTGATATC-3′ primers (Supplementary Fig. 2A). The *Tpi* gene marker (Supplementary Fig. 2B) was also used to amplify single nucleotide polymorphisms (SNPs) sites (e4183) using the *Tpi*_282F, 5′-GGTGAAATCTCCCCTGCTATG-3′ and *Tpi_*850R, 5′-AATTTTATTACCTGCTGTGG-3′ primer pair, which resulted in approximately 500-bp PCR fragment. The PCR products for primers *Tpi*_282F and *Tpi*_850R were sequenced with primer *Tpi*_412F, 5′-CCGGACTGAAGGTTATCGCTTG-3′, to avoid overlap of two genes which may result in data ambiguity from heterozygous males due to insertion or deletion (indels) [19].

The *ace*-1 partial gene segment was amplified using primers set ace_1F (5′-GAACGCTGTCATGCTGTGG-3′) and *ace*_1R (5′-CCGAGGGCCATTCATTCACTC C-3′) designed [40], to produce a 972 bp fragment that was used to detect the SNP’s for resistance to organophosphate insecticides.

A PCR mixture of 16.0 μl of nuclease-free water (ThermoFisher Scientific, UK), 2.5 μl PCR buffer, 2.5 μl MgCl_2_ (2.5 mM), 0.5 μl dNTPs (10 mM), 1 μl of each primer (10 μM), 0.5 μl (2.5 units) of Dream Taq DNA polymerase® (ThermoFisher-Scientific) and 1 μl of the template DNA was used. The PCR thermo-cycling conditions for all the primer sets used in this study consisted of 94 °C (2 minutes), followed by 35 cycles of 94 °C (30s), 56 °C (35 s), and 72 °C (45 s) for denaturation, annealing, and extension, respectively. The PCR products were analyzed using electrophoresis in 1XTAE buffer on a 1.4% (w/v) agarose gel stained with 5 μl of ethidium bromide (0.5 μg/mL) and run at 80 V for 1 hour. The gels were then visualized under a UV light source and photographed using gel documentation system (U: GENIUS3-SYNGENE). The PCR products that gave positive results of the expected band sizes were then sequenced at Macrogen Europe BV. (Meiberg dreef 31, Amsterdam, the Netherlands).

### DNA polymorphism and comparative analysis

ClustalW was used to align trimmed (to 542 bp length fragment common to all *COI*A samples analyzed) sequences in MEGA-X ver. 10.1.7 [54]. *COI*A Sequences which were shorter than the selected 542 bp length were omitted from downstream analyses. The final list of 510 sequences was generated to enable DNA polymorphism analysis. To explore the biogeographic patterning of FAW and the relatedness of distinct sub-populations, we compared the diversity of the Ugandan FAW population with those of other geographic locations. For this, we separated the FAW population into four major groupings, each of which could be studied at the population level. The dataset was divided into four broad categories as 1. The rest of Africa; 2. America (which includes FAW from both North and South America); 3. Asian countries (which includes populations from India, Bangladesh, China, Korea, Vietnam, Japan, Myanmar, and Pakistan); and 4 Uganda based on the geographical distribution. These groups were analyzed for descriptive statistics such as nucleotide diversity, number of haplotypes (H), haplotype diversity (Hd), genetic neutrality tests and mismatch distribution analysis using DnaSP ver 6.12.03 [55]. We analyzed the previously reported sequences [44] (for polymorphisms) from America (*n* = 99), Asia (India, China, Japan, Vietnam, Korea, Bangladesh, Pakistan, Myanmar) (*n* = 178), and the rest of Africa (*n* = 146) in addition to the 87 samples from Uganda.

Out of the 95 *S. frugiperda* samples from Uganda, 92 were sequenced (for both the *COI* and *Tpi* genes), with five low-quality sequences being excluded from further analysis. As a result, only 87 samples were used for downstream analysis. The generated *COI* sequences revealed 100% similarity with *S. frugiperda* and were used to study the diversity of Ugandan populations*.* Additional 423 Genbank sequences from different parts of the world, and previously used by [44] were also retrieved for comparative sequence analysis and inter-population studies. Details and approximate collection sites of these FAW sequences from the Rest of Africa (n = 146), America (n = 99), and Asian nations (India, China, Japan, Vietnam, Korea, Bangladesh, Pakistan, Myanmar) (n = 178), are reported by [44]. For molecular identification of *S. frugiperda* strains, we investigated the Ugandan *S. frugiperda COI* and *Tpi* gene polymorphisms using strain defining loci and polymorphic sites which were previously reported [18, 20, 23, 24]. The *mCO*I602 strain defining polymorphic locus is found in the barcode region (*COI*A) (Supplementary Fig. 2A), and has been used to discriminate between “C” and “R” strains of FAW in both the Western Hemisphere and other areas of the world [24, 36, 41]. Another strain marker segment (*COI*B) was studied, which contains polymorphic loci *mCO*I1164D and *mCO*I1287R, which is used to determine the five haplotypes, four of which belong to the Corn strain (CSh1–4) and one to the Rice Strain category (Supplementary Fig. 2A), and further confirm FAW species host strain identity. The *Tpi* partial gene segment was also used to determine the identity of the *S. frugiperda* host strain, and the results were compared to the *COI* marker. The *S. frugiperda* host strains were identified with the presence of either nucleotide base letter “C” or “T”, at g*Tpi*183, for corn-strain (*Tpi-C*) or rice- strain (*Tpi-R*), respectively.

### Haplotype network and phylogenetic comparisons

The popART software (*Ver.* 1.7) [56] was used to infer a haplotype network graphic using a median joining network method described for inferring haplotype relationships [57]. To infer the evolutionary relationship between Ugandan sequences, and retrieved sequences (out-group), other *Spodoptera* species, and *S. frugiperda* corn and rice strains) from NCBI [58], which were previously reported by [36, 41], we generated neighbor-joining consensus tree using Jukes-Cantor genetic distance model with 70% support threshold and 1000 number of replicates in a bootstrap resampling method using Geneious prime (*ver* 2021.2.2.) [59]. Both *COI* and *Tpi* DNA sequences of FAW selected were compared with DNA sequences of the FAW host strains and other *Spodoptera* species that are available in the GenBank [58].

### Analysis of molecular variance (AMOVA)

Analysis of molecular variance was used to investigate the genetic structure of the four geographical groups. We conducted six independent AMOVA analyses, first comparing the four geographical groups and then comparing alternative groupings combinations (i.e. Uganda and Asia, Uganda and America, Rest of Africa and Asia, Rest of Africa and America, Asia and America). These analyses were performed using the GenAlEx software *ver*. 6.5 [57, 58] using 9999 permutations. The order for this analysis was based on four predetermined geographical regions America, Rest of Africa, Asia and Uganda. The percentage of observed variance within and between populations was calculated initially among all the regions and then the same analysis was performed between different combinations of two groups (i.e. Uganda and Asia, Uganda and America, Rest of Africa and Asia, Rest of Africa and America, Asia and America).

### Detection of SNPs associated with organophosphate resistance in *S. frugiperda*

We investigated six mutations previously identified to be involved in resistance to organophosphates. Three of these mutations, A201S, G227A, and F290V (numbering corresponding to *Torpedo californica* mature enzyme) [39] in the *ace*-1 gene have been reported to confer resistance to organophosphate insecticides in several other insects [37, 38, 46–48, 52–57, 60]. Later, these mutations were detected in FAW strains which are resistant to organophosphates [4, 16, 39].

The electro-pherograms obtained were visualized, edited, and assembled with Chromas software package (Version 2.6.6). The two reference sequences from the GenBank— KC435024 and KC435023 for resistance and susceptibility, respectively—were aligned alongside the Ugandan nucleotide sequences. MEGA 7.0 Clustal X [54] was used for the alignment. Following the alignment of the sequences, searches were made at the SNP locations g-301, g-565, g-380, g-396, g498, and g-768. The presence of particular nucleotides at particular mutation sites—namely, g-301 = G/T, g-380 = G/C, g-565 = T/G, g-396 = G/A, g-498 = A/G, and g-768 = C/G—was then used to identify the SNPs [16, 39]. A mutation associated with organophosphorus pesticide resistance is denoted by the second letter option in each SNP position [40].

### Statistical data analysis

Using R software version 3.5.3 (R Core Team, 2019), bar plots were created to show the pattern of strain distribution for *S. frugiperda* populations in Uganda based on mitochondrial gene *COI* and *Tpi* gene markers.

## Supplementary Information

**Additional file 1: Supplementary figure 1.** Amplification of *Spodoptera frugiperda cytochrome oxidase 1* subunits, *Triosephosphate isomerease* and *acetylcholinesterase* partial gene segments. **Supplementary figure 2.***CO*I and *Tpi* gene segments used in this study.

## Data Availability

The datasets generated and/or analyzed during the current study are available in the National Center for Biotechnology Information (NCBI) repository. The sequencing data generated and used in this study are designated by the GenBank accession numbers OP020715, OP020716, OP020881 to OP020883, and OP185931 to OP185937.
