# Supplementary material for: Genetic analyses and detection of point mutations in the acetylcholinesterase-1 gene associated with organophosphate insecticide resistance in fall armyworm (Spodoptera frugiperda) populations from Uganda
Source: BMC Genomics. 2023 Jan 16;24:22. doi: 10.1186/s12864-022-09093-4 (PMC9841645; doi:10.1186/s12864-022-09093-4)
Supplement: Supplementary file 1 — Additional file 1: Supplementary figure 1. Amplification of Spodoptera frugiperda cytochrome oxidase 1 subunits, Triosephosphate isomerease and acetylcholinesterase partial gene segments. Supplementary figure 2.COI and Tpi gene segments used in this study. [file 12864_2022_9093_MOESM1_ESM.docx]

**PCR amplification of *S. frugiperda* partial gene segments**


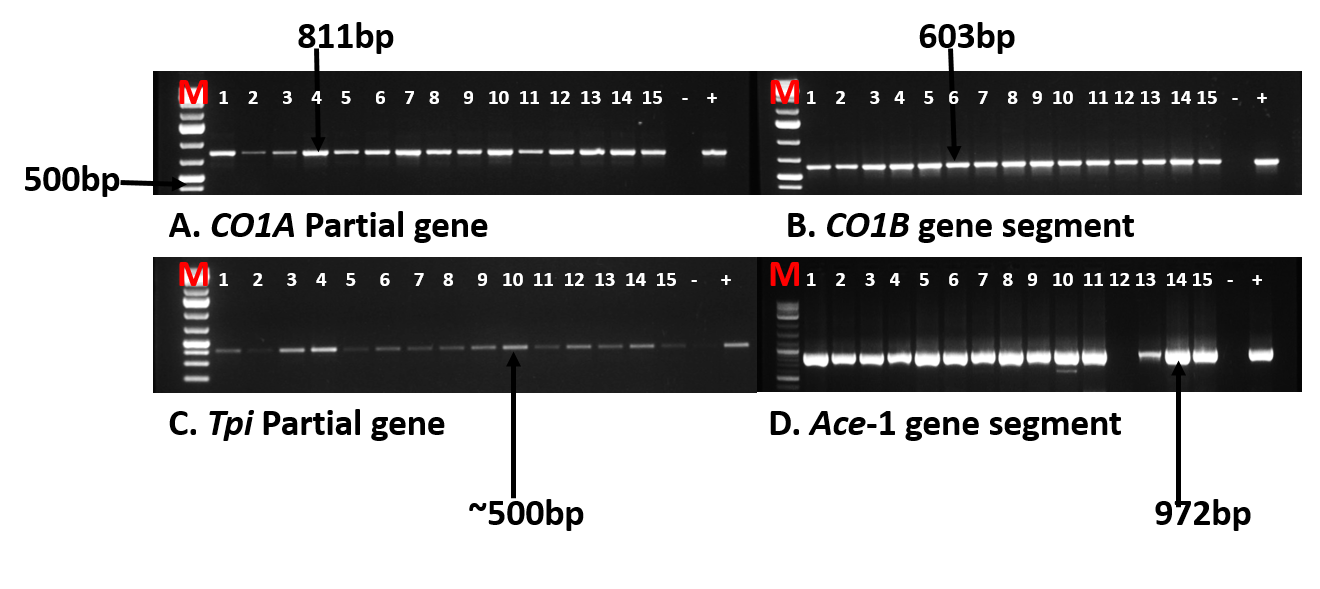


Supplementary figure 1. Amplification of *Spodoptera frugiperda cytochrome oxidase 1* subunits, *Triosephosphate isomerease* and *acetylcholinesterase* partial gene segments. (A and B)-mitochondrial *Cytochrome oxidase* 1A and B respectively. (C)- *Triosephosphate isomerase* sex-linked nuclear gene, and (D) - the *Ace-*1 gene segment. M = 1kb+ DNA ladder, (**-**) = Negative control, (**+**) = Positive control and 1-15 are representative samples of *Spodoptera frugiperda.* A, B, C, and D partial gene segments were amplified using *CO*I_101F/*CO*I_911R; *CO*I_891F/*CO*I 1472R; *Tpi*_282F/*Tpi* 850R and *ace_*1F/*ace*_1R primer pairs, respectively. Cropped gel images were extracted from the full length original gels (1, 2, 3, and 4 for A, B, C and D respectively).

**Uncropped full length original gels**

1. **Mitochondrial *Cytochrome oxidase* 1A amplified with *CO*I_101F/*CO*I_911R primer pair**


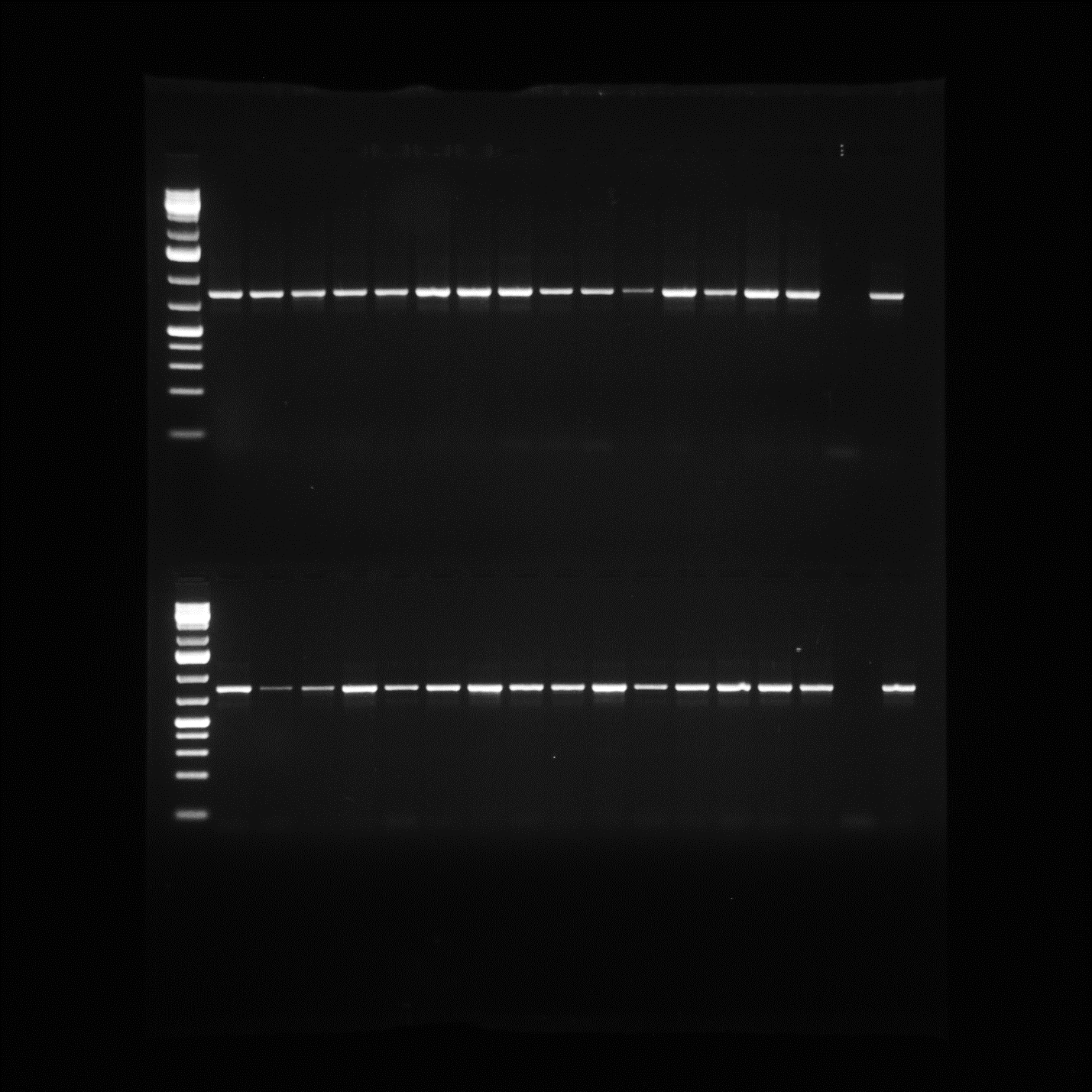


Uncropped Supplementary figure 1A: mitochondrial *Cytochrome oxidase* 1A of *Spodoptera frugiperda.* Expected band size=811bp

1. **Mitochondrial *Cytochrome oxidase* 1B amplified with *CO*I_891F/*CO*I 1472R primer pair**


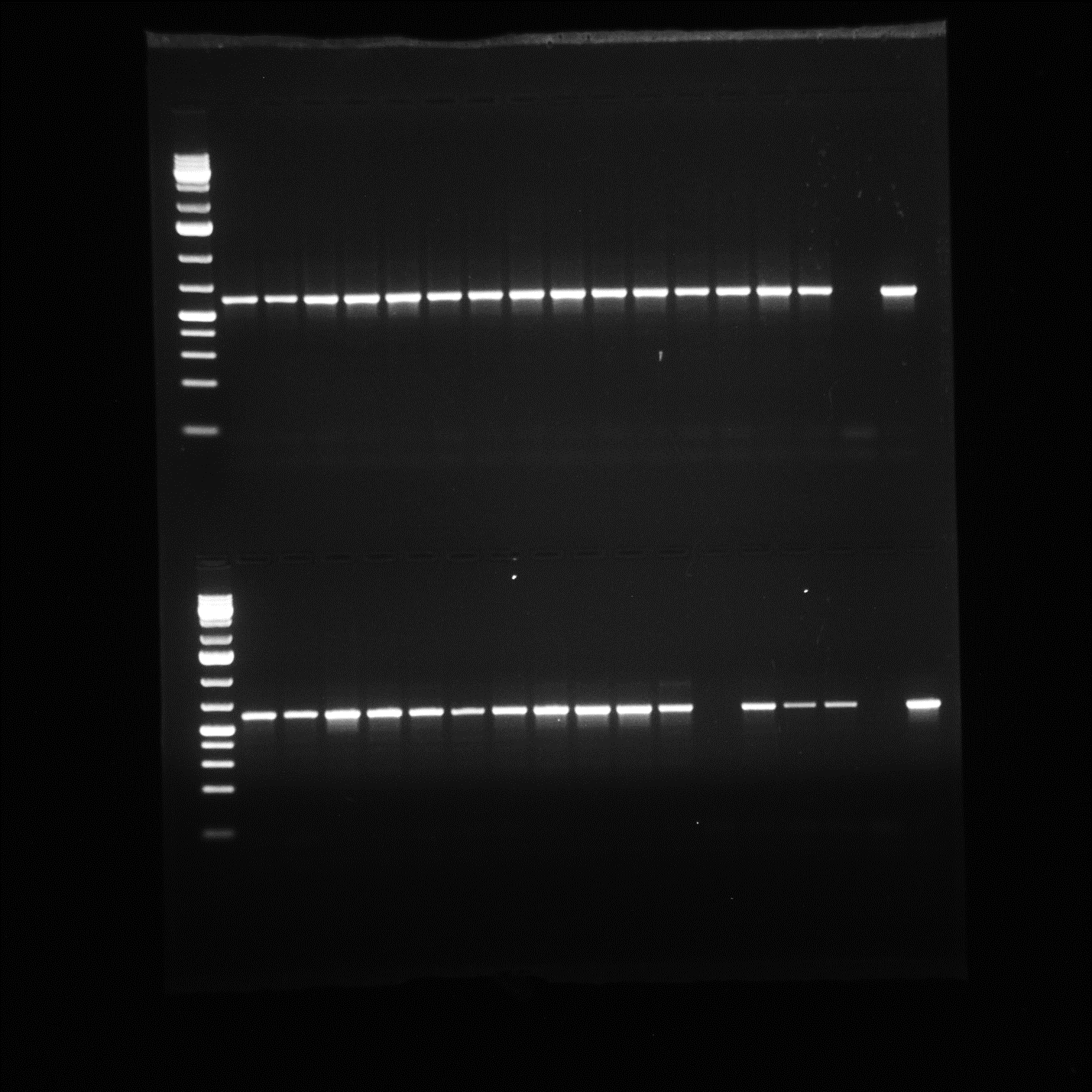


Uncropped Supplementary figure 1B: mitochondrial *Cytochrome oxidase* 1B of *Spodoptera frugiperda.* Expected band size=603bp

1. ***Triosephosphate isomerase* (Tpi) amplified with *Tpi*_282F/*Tpi* 850R primer pair**


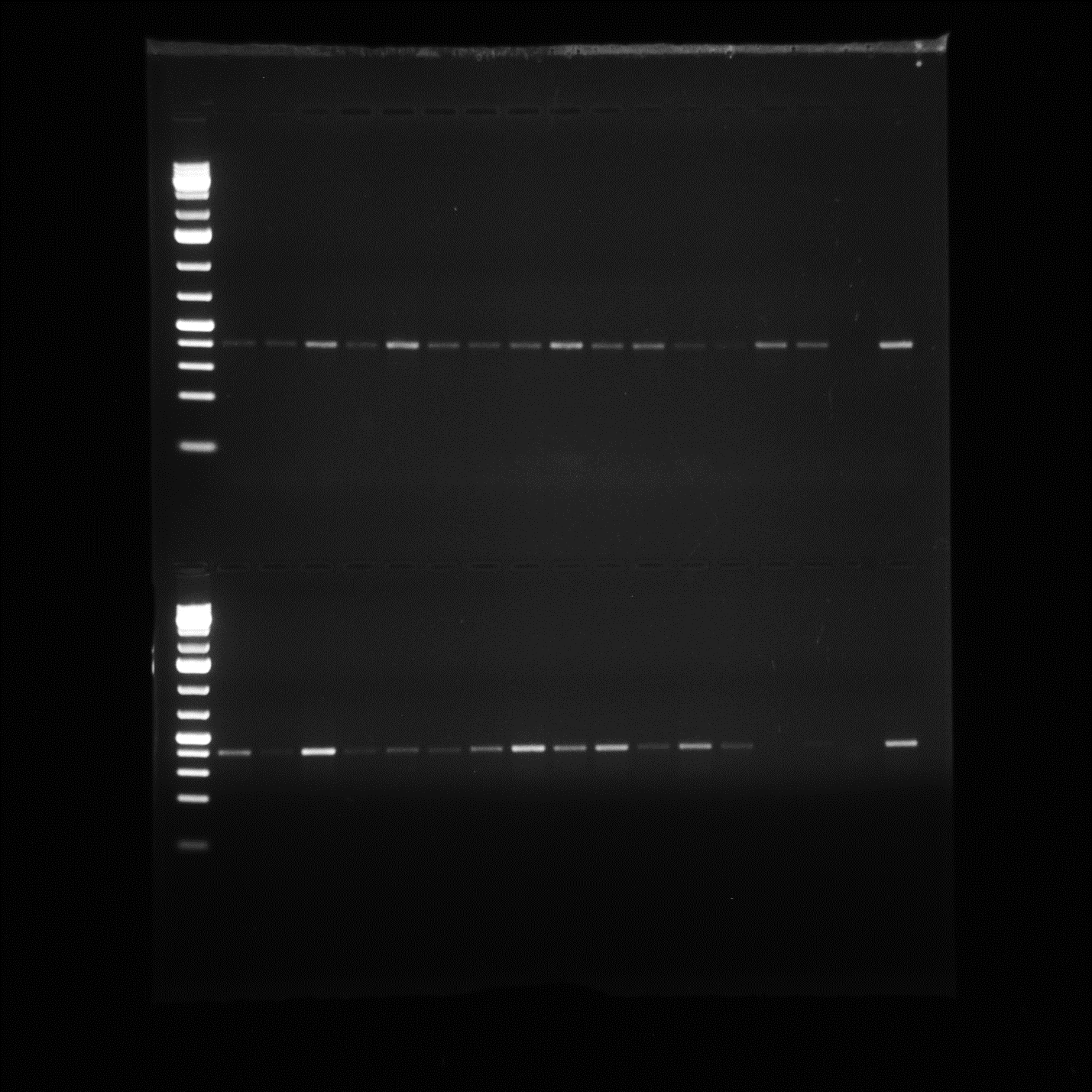


Uncropped Supplementary figure 1C: *Triosephosphate isomerase* (Tpi) sex-linked nuclear gene of *Spodoptera frugiperda.* Expected band size=~500bp.

1. ***Acetylcholinesterase-1(Ace-*1) *partial* gene amplified with *ace_*1F/*ace*_1R primer pair**


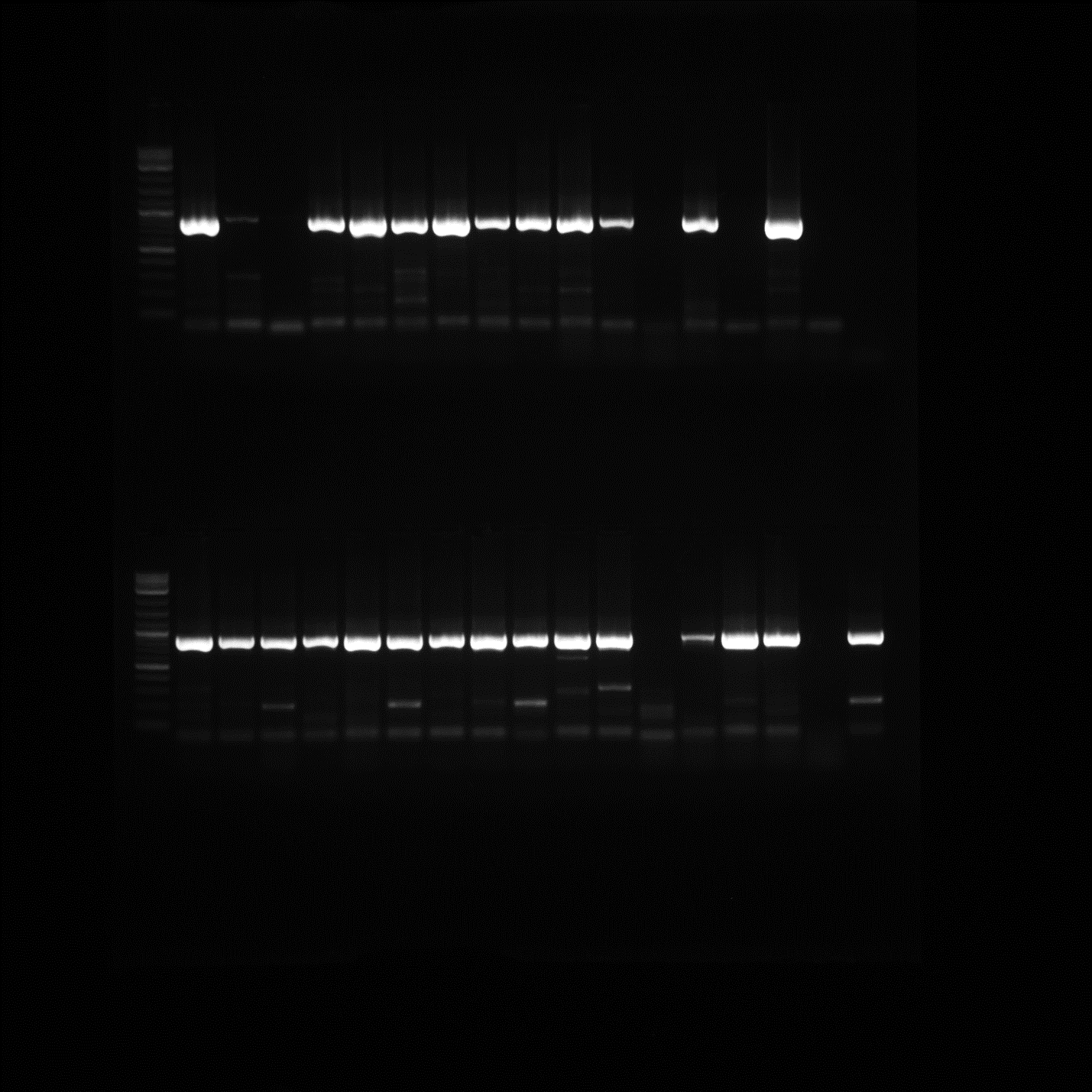


Uncropped Supplementary figure 1D: *Acetylcholinesterase-1(Ace-*1) *partial* gene segment of *Spodoptera frugiperda.* Expected band size=972bp


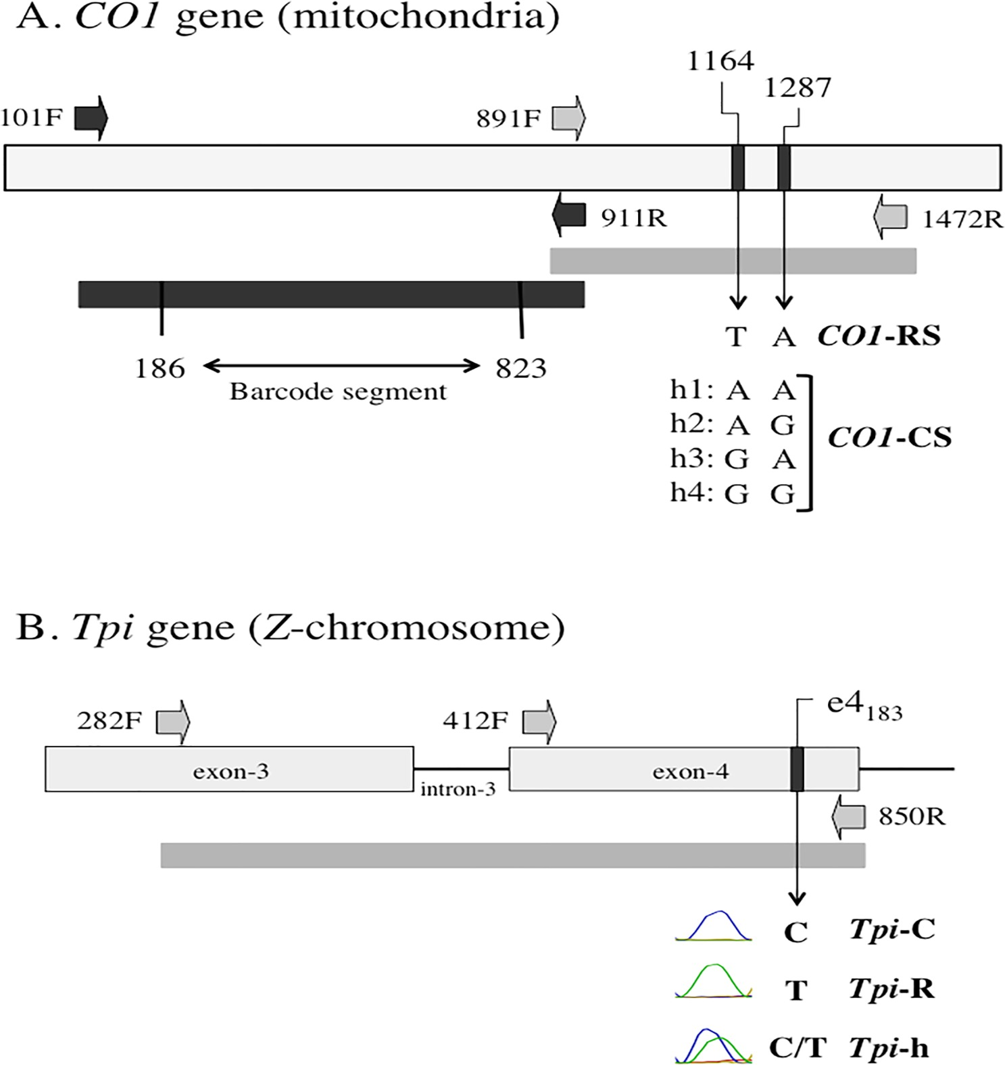
**Picture source: “(Rodney et al., 2017)**(18)**”**

**Supplementary figure 2. *CO*I and *Tpi* gene segments used in this study. A:***CO*I gene segment. PCR primers 110F/911R (Dark block arrows) were used to amplify the barcode region (dark bar). Primers 891F/1472R (Grey block arrows) amplified *CO*I*_1164_* and *CO*I*_1287_* polymorphic sites fragment (light bar).  The Presence of nucleotide base “T” at *CO*I*_1164_* together with a nucleotide base “A” at *CO*I*_1287_* defines *CO*I-RS. Four corn-strain (*CO*I-CS) haplotypes (h1-h4) exist. They are defined by nucleotide base “A” or “G” at both *CO*I_1164_ and *CO*I_1287_. **B:** Portion of the fall armyworm *Tpi* gene. Block arrows indicate PCR primers (282F/850R).
